# The Contribution of Arterial Calcification to Peripheral Arterial Disease in Pseudoxanthoma Elasticum

**DOI:** 10.1371/journal.pone.0096003

**Published:** 2014-05-06

**Authors:** Georges Leftheriotis, Gilles Kauffenstein, Jean François Hamel, Pierre Abraham, Olivier Le Saux, Serge Willoteaux, Daniel Henrion, Ludovic Martin

**Affiliations:** 1 PXE Health and Research Center, University Hospital of Angers, Angers, France; 2 UMR CNRS 6214 - Inserm 1083, School of Medicine, l'UNAM University, Angers, France; 3 Department Cell and Molecular Biology, John A. Burns School of Medicine, University of Hawaii, Honolulu, Hawaii, United States of America; 4 UPRES 3860, School of Medicine, l'UNAM University, Angers, France; 5 Clinical Research Center, University Hospital of Angers, Angers, France; Medical University of Graz, Austria

## Abstract

**Background and aims:**

The contribution of arterial calcification (AC) in peripheral arterial disease (PAD) and arterial wall compressibility is a matter of debate. Pseudoxanthoma elasticum (PXE), an inherited metabolic disease due to *ABCC6* gene mutations, combines elastic fiber fragmentation and calcification in various soft tissues including the arterial wall. Since AC is associated with PAD, a frequent complication of PXE, we sought to determine the role of AC in PAD and arterial wall compressibility in this group of patients.

**Methods and Results:**

Arterial compressibility and patency were determined by ankle-brachial pressure index (ABI) in a cohort of 71 PXE patients (mean age 48±SD 14 yrs, 45 women) and compared to 30 controls without PAD. Lower limb arterial calcification (LLAC) was determined by non-contrast enhanced helicoidal CT-scan. A calcification score (Ca-score) was computed for the femoral, popliteal and sub-popliteal artery segments of both legs. Forty patients with PXE had an ABI<0.90 and none had an ABI>1.40. LLAC increased with age, significantly more in PXE subjects than controls. A negative association was found between LLAC and ABI (r = −0.363, p = 0.002). The LLAC was independently associated with PXE and age, and ABI was not linked to cardiovascular risk factors.

**Conclusions:**

The presence of AC was associated with PAD and PXE without affecting arterial compressibility. PAD in PXE patients is probably due to proximal obstructive lesions developing independently from cardiovascular risk factors.

## Introduction

Arterial calcification (AC) is an independent risk factor for cardiovascular disease [1], including peripheral arterial disease (PAD) [2]. The role of arterial calcification in PAD is unclear but is related to the site and location of calcification within the arterial layers. Intimal calcification is frequently associated with atherosclerosis, whereas calcification of the medial layers is frequently associated with aging or acquired metabolic diseases (such as diabetes and chronic renal insufficiency) and usually associated with arterial stiffening [3]. Arterial calcification has also been reported in young patients with a range of inherited monogenic diseases as a result of impaired regulation of soft tissue calcification [4]. Pseudoxanthoma elasticum (PXE; OMIM 264800, prevalence 1/25000 to 1/50000) is an autosomal recessive multisystem disease resulting from mutations in *ABCC6* that encodes an ATP-binding cassette transporter mainly expressed in the liver and kidneys [5], [6]. This disease is mainly characterized by progressive calcification of the elastic fibers in the skin, retinal Bruch's membrane and the medial layers of large and medium sized peripheral arteries. PXE patients therefore seem to develop cardiovascular events and complications, including PAD, at a younger age than the normal population, although without obvious cardiovascular risk factors [7]–[11]. Using the ankle-brachial index, an index screening for PAD, our group recently confirmed a high prevalence of PAD in a large cohort of PXE [12]–[13]. Unexpectedly, our findings revealed preserved arterial wall compressibility despite the presence of medial calcification in PXE, suggesting that AC might not play a direct role in the development of PAD in PXE. Since calcifications throughout the coronary and peripheral artery vasculature can be determined quantitatively and characterized using non-invasive computed tomography [14], we designed the study presented here to determine the association between AC in the lower limbs (LLAC) and PAD in PXE. The elective site of LLAC was also examined, as well as the impact of cardiovascular risk factors in the calcification process and PAD.

## Materials And Methods

### Study population

Seventy-one consecutive PXE patients were enrolled in this study at the PXE Consultation Center of the University Hospital of Angers, France. The diagnosis of PXE was based on a combination of established criteria for indisputable PXE, including clinically suggestive skin lesions, angioid streaks and histologically proven fragmented and calcified elastic fibers on skin biopsy [15]. A control group (n = 30) matched for gender and without PAD or cardiovascular events was also recruited for the study.

Medical history, age, body mass index and smoking habits were recorded for all participants. Previous cardiovascular events (stroke and myocardial infarction) and the presence or absence of cardiovascular risk factors were defined according to the following criteria: 1) Hypertension: systolic (SABP)>140 mmHg and diastolic (DABP) >90 mmHg blood pressure and/or use of anti-hypertensive medication, 2) Diabetes: fasting blood glucose ≥7.0 mmol/l (≥126 mg/dL) and/or use of glucose-lowering medication including insulin, and 3) dyslipidemia: low density (LDL) cholesterol >3.4 mmol/l, high density (HDL) cholesterol lipoproteins <1 mmol/l, triglyceridemia >2 mmol/l and/or use of lipid-lowering medication. Blood samples were collected after overnight fasting for measurement of total cholesterol, LDL, HDL, blood glucose and glycosylated hemoglobin using standard laboratory techniques. Brachial systolic (SABP) and diastolic (DABP) blood pressure were measured in the supine position with an automatic sphygmomanometer (Welch Allyn Inc., Model SPOT, France). Cardiovascular risk stratification of the patients and controls was performed according to the Framingham model [including age, diabetes, smoking, SABP, total and HDL-cholesterol] modified for European populations (F-L score in %) [16], and diabetic patients were excluded from the study. The whole study group was subdivided into three categories according to the Framingham score: high (>20%), intermediate (10–20%) and low (<10%) risk of cardiovascular events in the next 10 years. (See http://www.framinghamheartstudy.org/risk-functions/cardiovasculardisease/10-year-risk.php for further details). All patients gave informed written consent and the study was approved by our local ethics committee (CPP Ouest II – Angers - France) and registered at ClinicalTrials.gov (Identifier #: NCT01446393).

### Detection of lower limb peripheral arterial disease

For all participants, the ABI was measured with a bidirectional 8-MHz ultrasound Doppler velocimeter and a pneumatic sphygmomanometer according to standard methods [17]. Ankle systolic pressure was measured in the posterior tibialis and dorsalis pedis arteries of both legs and in the brachial artery of both arms. Determination was performed at least twice for each artery. The ABI was calculated separately for each leg using the highest ankle systolic pressure as the numerator and the highest brachial systolic pressure as the denominator, according to the latest recommendations [13]. The lower of the left or right ABI was considered for determination of PAD. The ABI was considered normal between 0.91 and 1.39 and the presence of PAD was based on an ABI ≤0.90. An ABI >1.40 was suggestive of PAD with non-compressible arterial wall.

### Calcium scoring in the lower limb arteries

All participants underwent a non-contrast enhanced 64-row multidetector-computed tomography scan (Brillance 64, Philips HealthCare, Dest, The Netherlands) of the lower limbs, from the iliac crest to the tips of the toes, without injection of contrast medium. All scans were performed according to a standardized protocol including 64×0.625 collimation, at 120 kV with intensity (in mA) adjusted according to the patient's BMI and dose modulation along the Z axis. One millimeter thick slices were reconstructed every 0.7 mm. Calcium scoring was performed using automated 3D image analysis software (Synapse 3D, Fujifilm Medical Systems, Greenwood, SC, USA) with the investigators blind to the results of the patient's clinical status and ABI. Areas of calcification along the arterial path were identified automatically by the software at 4 pre-specified ranges of density (150, 200, 300 and 400 Hounsfield units, HU). Regions of interest were divided into 3 segments for each leg separately: 1) femoral (including the common and superficial femoral arteries) extending from the iliac crest to the opening of the adductor magnus, 2) popliteal (from the opening of the adductor magna to the origin of the anterior tibial artery) and 3) sub-popliteal (including the anterior and posterior tibialis and fibular arteries from their origin up to the malleolar region). The lengths of all segments were measured on the 3D scans and expressed in mm. Areas of calcification with a cross sectional area ≥0.7 mm^2^ and densities ranging between 150 and 400 HU were identified automatically on cross sectional images of the lower extremities. The arterial calcification score (Ca-score) was determined and expressed for each segment of both legs as the Agatston score [18]. The Agatston score of each segment was normalized to its length (HU.mm^−1^) to allow comparisons between each segment. Finally, an overall Ca-score was determined for each patient by adding together all segmental Ca-scores for both legs. Inter- and intra-observer variability in the determination of the Ca-score was evaluated from a randomly selected list of 10 scans by two different readers.

### Statistical analysis

All continuous variables are presented as mean ± SD. Categorical variables (expressed as %) were compared by Chi2 or exact Fisher's tests. The groups were stratified by age into the following categories: <39, 40 to 49, 50 to 59, and >60 yrs and divided into 2 groups according to the absence (i.e. a Ca-score = 0) or presence of LLAC (i.e. a Ca-score score >0). The patients of the PXE group were also stratified according to ABI (<0.90 or ≥0.90). Comparisons between the groups were performed using Kruskal-Wallis tests. Pairwise comparisons between the segmental LLAC scores in each group were performed using Wilcoxon's test for paired data. The confounding role of PXE status, age, gender, LDL cholesterol, smoking, menopausal status and arterial hypertension (independent variables) in LLAC was tested by logistic regression. Association of ABI with LLAC and CV risk factors was tested by logistic regression. All statistical analyses were performed with Stata 12.0 software (StataCorp, Texas, USA) and the level of statistical significance was set at P≤0.05 for all statistics.

## Results

The baseline characteristics of the study population are summarized in Table 1. There were more women (70%) than men among the PXE patients. Forty PXE patients had an ABI<0.90 and none had an ABI>1.40. Only one woman in the control group had an ABI<0.90. She was totally asymptomatic and without atherosclerosis on US examination.

**Table 1:** Demographic characteristics and lower leg calcification scores (LLAC) in patients with pseudoxanthoma elasticum (PXE) without (PAD−) or with (PAD+) peripheral arterial disease and controls.

	PXE_PAD−_ (n = 31)		PXE_PAD+_ (n = 40)			overall PXE		Controls (n = 30)		P (chi2)		
**Gender (m/f)**	6/25		15/25			21/50		9/21		0.263		
**Hypertension (%)**	6		40			25		11		0.002		
**Dyslipidemia (%)**	6		47			30		14		0.000		
**Smoking (%)**	26		27			27		18		0.637		
**Stroke (%)**	0		20			11		0		0.001		
**Myocardial infarction (%)**	3		15			10		0		0.048		
	mean	SD	mean	SD	PAD− vsPAD+	mean	SD	mean	SD	Ctrl vs PXE	Ctrl vs PXE_PAD−_	Ctrl vs PXE_PAD+_
**Age (years)**	43	13	52	13	0.006	48	14	43	14	0.007		0.011
**BMI (kg/m^2^)**	25	5	26	5		26	5	25	5	0.225		
**Cardiovascular risk score (%)**	1.5	2.1	3.5	3.7	0.002	2.6	3.2	2.3	4.2	0.003		0.008
**Systolic ABP (mmHg)**	118	11	125	15	0.012	122	14	126	17	0.034	0.05	
**Diastolic ABP (mmHg)**	73	10	73	11		73	10	77	14	0.025		
**Ankle-Brachial Index**	1.03	0.08	0.69	0.14		0.84	0.20	1.04	0.10	0.001		
**Blood glucose (mmol.L^−1^)**	4.87	0.58	5.19	0.85		5.05	0.76	5.18	0.67	0.089		
**Total Cholesterol (mmol.L^−1^)**	4.82	0.96	4.94	0.83		4.89	0.89	5.36	0.96	0.168		
**LDL (mmol.L^−1^)**	2.88	0.83	2.93	0.78		2.91	0.80	3.12	0.91	0.675		
**HBA1C (%)**	5.7	0.4	5.7	0.4		5.7	0.4	5.6	0.3	0.498		
**Arterial calcification (AC) scores**												
**LLAC (HU)**	2998	6145	9355	16137	0.003	6658	13188	335	879	<0.0001	0.006	<0.0001
**Femoral AC (HU.mm^−1^)**	5.08	12.04	16.93	25.69	<0.001	11.91	21.70	0.88	2.61	<0.0001		<0.0001
**Popliteal AC (HU.mm^−1^)**	1.63	5.11	5.03	15.01	0.045	3.59	11.91	0.10	0.53	<0.0001		<0.0001
**Sub-popliteal AC (HU.mm^−1^)**	3.38	7.00	9.44	22.05	0.025	6.87	17.50	0.15	0.40	<0.0001	<0.001	<0.0001

Hypertension was more frequent in PXE patients compared to controls. Dyslipidemia was not significantly more prevalent in PXE patients than controls, nor was smoking. The proportion of post-menopaused women was similar between the 2 groups (PXE = 48% vs controls  = 36%, p = 0.444). Seven PXE patients (10%) had a history of documented coronary artery disease, 6 of whom also had PAD. Eight had a history of ischemic stroke, all associated with PAD. Of the whole cohort, 85% of those with PXE patients and 81% of the controls were at low risk of cardiovascular disease, 12% of the PXE patients and 13% of the controls were at intermediate risk, while 2% of the PXE and 6% of the controls were at high risk of cardiovascular disease (p = 0.690 between groups).

### Lower limb arterial calcification

**Figure 1:**
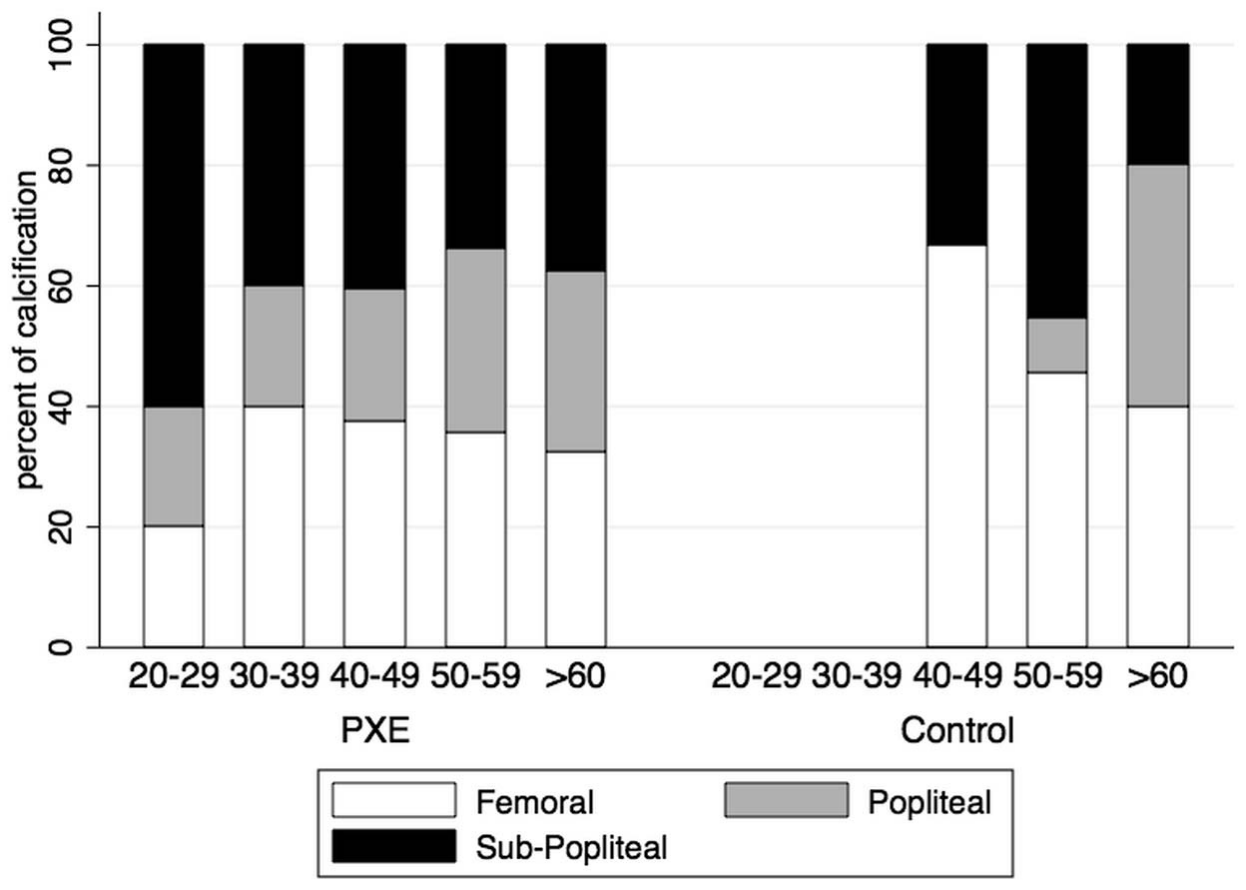
Prevalence of lower limb arterial calcification (LLAC) detected in arterial segments in pseudoxanthoma elasticum (PXE) and controls across age. Data are expressed as percentage of the total number of LLAC detected in a given age group.

**Figure 2:**
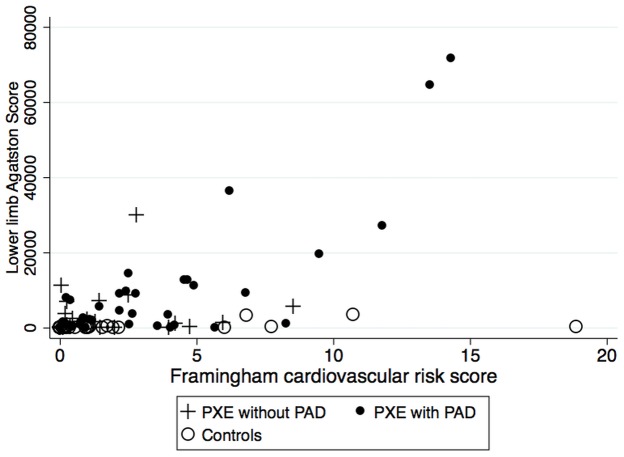
Relationship between Framingham cardiovascular risk score and calcification score in patients with pseudoxanthoma elasticum (PXE) and controls.

The intra-class correlation coefficient for inter-observer variability for determination of the calcification score was r = 0.998 (p<0.0001) and for intra-observer variability it was r = 0.964 (P<0.001). In both groups, the Ca-score increased significantly with age and in all segments (figure 1), and LLAC was detected in 95% of the PXE patients with PAD, 68% of the PXE patients without PAD and 32% of the controls. In both groups, the Ca-score was significantly higher in men (PXE: 15103±20521 HU and controls: 531±1029 HU) than in women (PXE: 2717±3951 HU, p = 0.0004 and controls  = 226±756 HU, p = 0.013 vs men). However, whatever the Ca-score, the prevalence of LLAC (i.e. the absence/presence of LLAC) was similar between PXE men (95%) and women (78%, p = 0.071) but significantly higher in control men (67%) compared to control women (18%,p = 0.015). Although the presence of LLAC was more frequent in the PXE than control women (91% versus 9%), there was no significant difference between the post-menopaused PXE (56%) compared to the controls (75%, p = 0.628) when LLAC were present (i.e. a Ca-score>0). In both groups, the Ca-score was significantly lower in the popliteal segment compared to the femoral and subpopliteal segments. The overall and segmental Ca-scores were significantly higher in PXE patients with PAD compared to PXE patients without PAD and the control group. The presence/absence of LLAC was independently associated with PXE status (OR = 0.06, CI = 0.02–0.20, p<0.0001) and the cardiovascular risk score (OR = 2.49, CI = 1.35–4.57, p = 0.003) (table 2) which was mainly due to age (OR = 1.12, CI = 1.05–1.19, p<0.0001). (figure 2).

**Table 2:** Logistic regressions between lower leg arterial calcification (dependent variable) and selected independent variables.

	Model 1	Model 2
	LLAC	LLAC
**PXE status**	−2.821	−3.292
	(4.25)**	(3.88)**
**F-L score**	0.911	
	(2.93)**	
**Age**		0.115
		(3.71)**
**Gender**		−2.310
		(2.53)*
**Smoking**		0.809
		(1.03)
**Hypertension**		1.161
		(0.91)
**LDL-C**		−0.039
		(0.09)
**Constant**	0.492	−1.527
	(1.25)	(0.83)

Absolute value of z statistics in parentheses.

*p<0.05;

**p<0.01.

ABI =  Ankle Brachial index, F-L =  Framingham-Laurier score, LLAC =  lower leg arterial calcification, LDL-C =  Low density lipoproteins.

### Relationship between LLAC and ABI

There was a weak but significant inverse relationship between ABI and Ca-score (r = −0.363, p = 0.002). The ABI was independently associated with the presence of LLAC (OR = 0.11 CI = 0.02–0.55, p = 0.007), but not with the cardiovascular risk score (OR = 0.83, CI = 0.67–1.03, p = 0.091) (table 3). A low ABI was associated with the presence of calcification in the femoral segment (OR = 0.079, CI = 0.011–0.55, p = 0.011) but not in the popliteal (OR = 1.40, CI = 0.32–6.16, p = 0.658) or subpopliteal segments (OR = 1.28, CI = 0.21–7.74, p = 0.791).

**Table 3:** Logistic regressions between ankle brachial index (dependent variable) and selected independent variables.

	Model1	Model2
	ABI	ABI
**LLAC>0**	−2.203	−1.819
	(2.68)**	(2.15)*
**F-L score**		−0.186
		(1.69)
**Constant**	1.609	1.726
	(2.08)*	(2.21)*

Absolute value of z statistics in parentheses.

*p<0.05;

**p<0.01,

ABI =  Ankle Brachial index, F-L score  =  Framingham-Laurier cardiovascular risk score, LLAC =  lower leg arterial calcification.

## Discussion

Since the pathophysiological role of LLAC in PAD is not fully understood, we decided to focus on PXE, an inherited condition in which AC is associated with a high prevalence of early PAD. We anticipated that this specific condition might disclose important information about the link between LLAC and PAD, independently from atherosclerosis.

### Patterns and distribution of arterial calcifications in PXE

Our quantitative analysis based on the CT-scan data confirmed a high prevalence of AC in PXE patients compared to the controls. There was no gender difference in PXE, in contrast to the controls where LLAC was more common in men. As expected, the menopausal status was characterized by a higher prevalence of LLAC [19] but it was not influenced by PXE. The prevalence of AC in our study was much higher than the prevalence of 31% reported in a previous semi-quantitative evaluation of AC based on standard X-ray [7], possibly due to the greater accuracy of CT scans in detecting smaller amounts of calcification. The amount of LLAC increased with age, although there was wide variability. It was barely detected in the controls before forty years of age but was found as early as twenty years of age in PXE patients (figure 1). Compared to other inherited calcifying diseases, the calcification process in PXE is generally considered to be slowly progressive [20], although extensive AC can develop in infancy [21]. The calcification process within the arterial segments of the legs was heterogeneous in both PXE and control groups, with AC predominating in the femoral and subpopliteal arteries (figure 1). In the general population AC is heterogeneously distributed across various vascular beds, predominating in the coronary arteries in men and in the aorta in women [22]–[24]. This heterogeneity is probably explained by the underlying pathophysiology, with calcification accumulating preferentially within the medial layers of the below-knee arteries in metabolic diseases [25] and within the intima layer of the proximal arteries, such as the femoral artery, where atheroma predominates [24], [26]. Since the spatial resolution of the non-contrast enhanced CT-scan was too low to discriminate between the intimal and medial deposition sites of AC, we can only speculate that in PXE, due to young age and the female predominance, AC develops earlier within the medial layers and later within the intima layer, when atheroma becomes obvious with aging. Moreover, the heterogeneous distribution of AC within the arterial segments of the same limb suggested differences in the local balance in anti- and pro-calcifying factors between the limb segments and joints. This could be explained by differences in the embryological origins of the arterial segments [27] and local rheological, hemodynamic and biomechanical strains in cyclic flexural stretching [28]. Interestingly, in contrast to the controls, the calcification process occurred simultaneously in all arterial segments in PXE patients, although the differences between the segments suggested that the calcification process in PXE is modulated at the local level.

### Contribution of arterial calcifications to PAD in PXE

Since PAD and AC are both associated with PXE, we examined the contribution of AC to PAD in this context. In agreement with previous studies [9], [29], we observed a high prevalence (44%) of PXE patients with a low ABI at rest. Although ABI was associated with the presence of LLAC, the negative relationship with LLAC was unexpected. In fact, calcium deposition within the arterial wall and fragmentation of elastic fibers [30] were expected to reduce arterial wall compressibility to the pressure exerted by the pneumatic cuffs and increase ABI (i.e. a positive relationship with LLAC) [31], [32]. We believe that there are no reports in the literature of ABI >1.40 in other PXE cohorts [7], [33] except when associated with diabetes [12]. Reduced compressibility of the arterial wall is generally associated with aging [34] and acquired metabolic diseases such as diabetes [35], [36], CKD [37] [38] or Monckeberg's atherosclerotic disease [38], [39]. The impact of calcification and elastic fiber fragmentation on arterial stiffness in PXE is unclear, some authors reporting a tendancy to increased arterial stiffness [33], and others reduced stiffness [30], [40] or increased wall compressibility [41]. Our findings together suggest that AC in PXE is not accompanied by increased stiffness of the arterial wall. This conclusion needs to be viewed with caution since an association between AC and PAD has not been clearly established in other calcifying diseases [13], [31]. In diabetics with incompressible arteries (i.e. ABI>1.40), a poorer prognosis has been reported to be linked to obstructive arterial disease compared to those with mediacalcosis alone [42]. The low ABI in our study was preferentially associated with calcification of the femoral segment, suggesting that the drop in peripheral pressure is linked to the development of an arterial obstruction in the proximal arterial segments rather than calcification in the distal arteries. This finding is in accordance with the fact that ischemia requiring surgery often occurs in PXE patients suffering from proximal lesions [43], [44]. Although the pressure drop below a proximal stenosis can be the most probable explanation to the low ABI, changes in arterial stiffness and compliance, and arterial wave reflection should be considered, but were not measured in the present study. We described recently the vascular phenotype of a murin model of PXE (Kauffenstein et al. ATVB in press). Noteworthy, these animals displayed scattered arterial calcium deposition with a lower elasticity and increased myogenic tone. Such changes may be a consequence of metabolic changes associated to the ABCC6 deficiency and could also contribute to our findings.

### Contribution of CV risk factors to AC and PAD in PXE

Our findings showed wide variations in the amount of AC, which increased with age and suggested that confounding factors contribute to the severity of the calcification process in PXE [45]. The common vascular risk factors should be considered first as potential contributors to the severity of both AC and PAD in PXE. A large majority of the PXE patients studied were at low risk for CV events according to the Framingham equation, although those with PXE and PAD were at higher risk of CV events since they were mostly older men. In accordance with recent findings, aging was the most prominent CV risk factor related to the severity of the calcification process [46]. On the other hand, the fact that ABI was not associated with CV risk factors suggests that PAD in PXE is not linked to common risk factors, but probably to a unique remodeling process.

### Study limitations

The cross-sectional design of this study did not allow us to determine the pattern progression of the calcification process and/or its potential impact on the overall cardiovascular morbidity-mortality of these patients. In contrast to the coronary vascular bed, where calcium scoring has been extensively studied using CT-scans, scoring of the calcification of the lower limb vessels has been less frequently evaluated [14] and a risk threshold remains to be determined. Despite this, our findings are consistent with the fact that AC is an early, slow process, taking several decades to develop, with wide inter-individual variability. In the absence of a validated risk threshold value for limb AC, the diagnosis and prognostic value of the calcification score remains to be determined prospectively in PXE.

## Conclusions

The present findings provide new information on the impact of calcification on arterial wall stiffness in PXE. Although calcification is a major phenotypic trait associated with PXE, it is likely that PAD results from complex remodeling occurring in proximal segments. Furthermore, both LLAC and PAD are differentially affected by local and systemic cardiovascular risk factors. Further studies are needed to determine the unique mechanisms leading to arterial occlusion in PXE.
